# L-3-n-Butylphthalide reduces ischemic stroke injury and increases M2 microglial polarization

**DOI:** 10.1007/s11011-018-0307-2

**Published:** 2018-08-16

**Authors:** Fangfang Li, Qingfeng Ma, Haiping Zhao, Rongliang Wang, Zhen Tao, Zhibin Fan, Sijia Zhang, Guangwen Li, Yumin Luo

**Affiliations:** 1 Institute of Cerebrovascular Disease Research and Department of Neurology, Xuanwu Hospital of Capital Medical University, 45 Changchun Street, Beijing, 100053 China; 2 Beijing Geriatric Medical Research Center and Beijing Key Laboratory of Translational Medicine for Cerebrovascular Diseases, Beijing, China; 3 Beijing Institute for Brain Disorders, Beijing, China

**Keywords:** Butylphthalide, Stroke, Microglia, Polarization, Inflammation

## Abstract

Overwhelming evidence suggests that microglia play an important role in ischemic injury and they polarize into two different phenotypes with distinct functions after ischemic stroke. We performed the present study to investigate whether L-3-n butylphthalide (NBP) has an effect on microglial polarization. Mice were subjected to transient middle cerebral artery occlusion (MCAO) for 45 min, and then immediately after reperfusion were treated with NBP or vehicle via the caudal vein for 7 consecutive days. 2,3,5-Triphenyltetrazolium chloride (TTC) staining showed that NBP treatment resulted in a tendency to decrease cerebral infarct volume at 1 day after MCAO, and significant decreased infarct volume at 3 days after MCAO. Sensorimotor function was evaluated by the adhesive removal test and balance beam test, which were superior in NBP-treated mice compared with vehicle-treated mice at 1 and 3 days after MCAO. Immunofluorescent staining further indicated that NBP treatment significantly increased the number of CD206^+^/Iba1^+^ M2 microglia/macrophages and reduced the number of CD16^+^/Iba1^+^ M1 cells at 3 and 7 days after MCAO reperfusion. Western blot also showed an elevation of M2 marker (arginase-1) in NBP-treated brains at 7 days after MCAO. In conclusion, our results clearly show that NBP treatment significantly mitigates ischemic brain damage and promotes recovery of neurological function in early phase after ischemic stroke, probably by skewing M1 microglia/macrophages polarization towards M2 phenotype. Thus, our study provides new evidence that NBP might be a promising candidate for ameliorating injury caused by ischemic stroke.

## Introduction

Ischemic stroke accounts for 85% of all stroke cases and is the second cause of global mortality closely following coronary heart disease (Mikulik and Wahlgren 2015). Without an effective intervention, ischemic lesions become irreversible in the first few hours after occlusion of blood flow. Moreover, because of lengthy rehabilitation is needed, a heavy burden is placed on family and society. Intravenous thrombolysis and mechanical thrombectomy are efficacious treatment strategies (Powers et al. 2018) and due to the narrow therapeutic time window, few patients can receive intravenous thrombolysis and mechanical thrombectomy. Thus, there is an urgent need to develop new drugs or methods to treat ischemic stroke in its early phase.

Inflammation plays an important role in the development of ischemic stroke, and microglia, as well as infiltrated macrophages play critical roles in regulating immune and inflammatory responses after brain injury (Perry et al. 2010). These cells are exquisitely sensitive to cerebral injury, changing morphology and switching to an activated phenotype following ischemic insult or other brain injury. Overwhelming evidence suggests that activated microglia/macrophages assume different phenotypes and exert different functions under specific microenvironmental signals (Hu et al. 2012). In particular, classically activated M1 microglia may exacerbate brain injury and be detrimental to neurological outcome by releasing inflammatory cytokines such as tumor necrosis factor (TNF-α), interleukin (IL)-12, IL-1β and IL-6(Girard et al. 2013). Meanwhile, alternatively activated M2 microglia may aid tissue repair and ameliorate brain injury by scavenging cell debris and secreting trophic factors that promote brain repair (Hu et al. 2015). Consequently, shifting activated microglial from the pro-inflammatory M1 phenotype towards the anti-inflammatory and tissue-reparative M2 phenotype offers a promising therapeutic target for the treatment of ischemic stroke.

L-3-n-Butylphthalide (NBP) is originally extracted from celery seeds, and was approved for the treatment of ischemic stroke by China Food and Drug Administration (CFDA) in 2002, and clinical studies have demonstrated its efficacy and safety (Xue et al. 2016). Mechanisms contributing to its efficacy include increasing axonal growth, promoting neurogenesis and neuroplasticity, alleviating oxidative stress, mitigating neuronal injury and suppressing apoptosis of neuronal cells (Sun et al. 2017; Yang et al. 2015; Zhang et al. 2016a; Zhao et al. 2014). Remarkably, NBP can also attenuate neuroinflammation in cultured astrocytes and microglia (Wang et al. 2013b; Wang et al. 2018a). However, the effect of NBP on microglial polarization after acute ischemic stroke has not been explored. In the present study, we examined the effect of NBP on microglial polarization and inflammatory responses in the middle cerebral artery occlusion (MCAO) model of ischemic stroke.

## Materials and methods

### Animal model

Adult male C57BL/6 mice (8–10 weeks, 20–25 g) were acquired from the Vital River Laboratory Animal Technology Co., Ltd. (Beijing, China). All animal experiments were approved by the Institutional Animal Care and Use Committee of Capital Medical University. Animals were housed at 20–23 °C with a 12 h light/dark cycle and a relative humidity of 50%. Food and water were available ad libitum. All attempts were made to reduce animal suffering and number of animals used. Animals were randomly divided into sham-operated group, vehicle-treated group and NBP-treated group (14 mg/kg/day). Mice were anesthetized with enflurane and the right common carotid artery (CCA), external carotid artery (ECA) and internal carotid artery (ICA) were exposed through a midline neck incision. A silicone rubber-coated monofilament suture (Catalogue number: 701956PK5Re; Doccol Corporation, Sharon, MA, USA) was inserted into the ICA via the ECA stump and slowly advanced to the middle cerebral artery (MCA). After occluding the right MCA for 45 min, the suture was removed to restore blood flow (reperfusion). Sham-operated mice were subjected to an identical surgical procedure without insertion of the suture. Regional cerebral blood flow was monitored by transcranial laser Doppler (LDF, PeriFlux System 5000; Perimed, Stockholm, Sweden). Successful occlusion was defined as a reduction of cerebral blood flow to approximately 20% of baseline. Successful reperfusion was defined as an increase of cerebral blood flow to approximately 70% of baseline. Rectal temperature was sustained at 37 ± 0.5 °C using a heating pad (CMA 150 Carnegie Medicine, Stockholm, Sweden). Mean arterial blood pressure was measured using a tail cuff during surgery, and arterial blood gases were monitored from 15 min after ischemia.

### Drug preparation and treatments

NBP (CSPC, NBP, Pharmaceutical Co., Ltd., Shijiazhuang, China) was dissolved with saline to a concentration of 1.4 mg/ml and NBP-treated mice were injected with NBP at a volume of 0.1 mL/10 g (14 mg/kg/day) through caudal vein immediately after reperfusion for 7 consecutive days. Injection time points were 0, 1, 2, 3, 4, 5, 6 and 7 days post MCAO. Sham-operated mice and vehicle-treated mice received the same volume of saline as NBP-treated mice.

### Measurement of infarct volume

Mice were decapitated at 24 h and 72 h after cerebral ischemia/reperfusion and brains were then rapidly removed. The brains were cut into 1 mm-thick coronal slices and dyed with 2% 2, 3,5-triphenyltetrazolium chloride (TTC), as previously described (Suenaga et al. 2015). The infarct volume was calculated as follows: V% = ∑(A_1_ + A_2_ + A_3_ + … + A_n_)/ ∑area of the contralateral hemisphere, where An = area of the contralateral hemisphere – non-infarct area of the ipsilateral hemisphere. Data were measured using Image J (National Institute of Health, Bethesda, MD, USA) by a person blinded to the experiment design.

### Measurement of neurological deficits

Sensorimotor function was evaluated before MCAO, as well as at 1 and 3 days after stroke using adhesive removal test, as described in previous studies (Liu et al. 2017). Briefly, the mouse was placed in a cage and an adhesive tape (30mm^2^) was pasted onto the distal radial section of the left forelimb as a tactile stimulus. Time to perceive and time to remove the tape was both recorded. Each animal was examined three times with a maximum time of 120 s per test.

The balance beam test was used to evaluate motor and balance coordination capacity. The general protocol was as follows: mice were placed on a balance beam (length, 100 cm; width, 2 cm; elevation, 40 cm) and a 6-point rating scale was adopted to assess their locomotor ability (Wang et al. 2017). Each single test lasted a maximum of 2 min and the procedure was conducted before, as well as 1 and 3 days after MCAO. Both behavioral tests were conducted by a person blinded to the experimental design.

### Terminal transferase dUTP nick end labeling (TUNEL) assay

Apoptosis of neurons was evaluated by co-labeling NeuN (1:100, Millipore, Danvers, MA, USA) and TUNEL (in situ cell death detection kit, FITC Roche, Mannheim, Germany), in accordance with the manufacturers’ protocols. Three visual fields in the ipsilateral cortex were randomly selected at 200× magnification and three slides were analyzed from each brain. Number of TUNEL^+^/NeuN^+^ double positive cells was manually counted in each brain section.

### Immunofluorescence staining

Brain slices were obtained and used for immunofluorescence staining, as previously described (Ma et al. 2016). The primary antibodies were goat anti-CD206 (1:200; R&D Systems, Minneapolis, MN, USA), rat anti-CD16/32 (1:100; BD Pharmingen, San Jose, CA, USA) and rabbit anti-ionized calcium-binding adapter molecule 1 (Iba1) (1:200; Wako Pure Chemical Industries, Osaka, Japan). The fluorophore-conjugated secondary antibodies (Invitrogen Corporation, Carlsbad, CA, USA) used were donkey anti-goat antibody conjugated to Alexa 488 (1:200), donkey anti-rat antibody conjugated to Alexa 594 (1:200) and goat anti-rabbit antibody conjugated to Alexa 594 (1:200). Images were captured using a fluorescence microscope (Carl Zeiss, Oberkochen, Germany). Mean merged cell counts were calculated from three randomly selected microscope fields in the cortex of each section, and three consecutive sections were analyzed from each brain. All images were manually counted. Data are expressed as mean number of cells/mm^2^. The person evaluating TUNEL staining and immunofluorescence staining was blinded to the treatment group of the mice.

### Western blot analysis

Ipsilateral cerebral tissue was homogenized in radioimmunoprecipitation assay (RIPA) lysis buffer containing protease inhibitors (aprotinin, leupeptin, phenylmethylsulfonyl fluoride, and pepstatin) and phosphatase inhibitors (Sigma cocktail; Sigma Aldrich, St. Louis, MO, USA). After sonication, protein concentration was determined and sodium dodecyl sulfate–polyacrylamide gel electrophoresis (SDS-PAGE) was then performed. Nitrocellulose membranes were incubated with anti-arginase-1(Arg1) (1:500; 9819, Cell Signaling Technology, Boston, MA, USA) and β-actin (1:1000; 1616, Santa Cruz Biotechnology Inc., Santa Cruz, CA, USA) at 4 °C overnight, followed by corresponding horseradish peroxidase (HRP)-conjugated secondary antibodies (1:5000; Santa Cruz Biotechnology Inc.). Finally, immunoblots were visualized using an enhanced luminescence kit (Millipore, Billerica, MA, USA). Blot intensities were then semi-quantified using Image J software (National Institute of Health), with β-actin as an internal control. Blot intensities were also examined in a blinded manner.

### Statistical analysis

Data were analyzed with SPSS Statistics 21.0 software (IBM Corp., Armonk, NY, USA). All results are presented as mean ± SEM. Comparison between two groups were analyzed using Student’s two-tailed *t*-test, while comparisons across multiple groups were analyzed by one-way analysis of variance (ANOVA) followed by Tukey’s post-hoc test. Differences among multiple groups with multiple measurements over time were analyzed using two-way repeated measures ANOVA. All tests were considered statistically significant at *p* < 0.05.

## Results

### NBP treatment significantly reduces infarct volume at 3 days after MCAO

In this study, a total of 118 animals were used for TTC staining (23 mice), behavior tests (22 mice), and immunofluorescence staining,western blot or TUNEL staining (73 mice). Mortality of vehicle-treated mice was 21.3%, and the mortality of NBP-treated mice is 15.9%. There was no significant difference in mortality between the two groups.

**Fig. 1:**
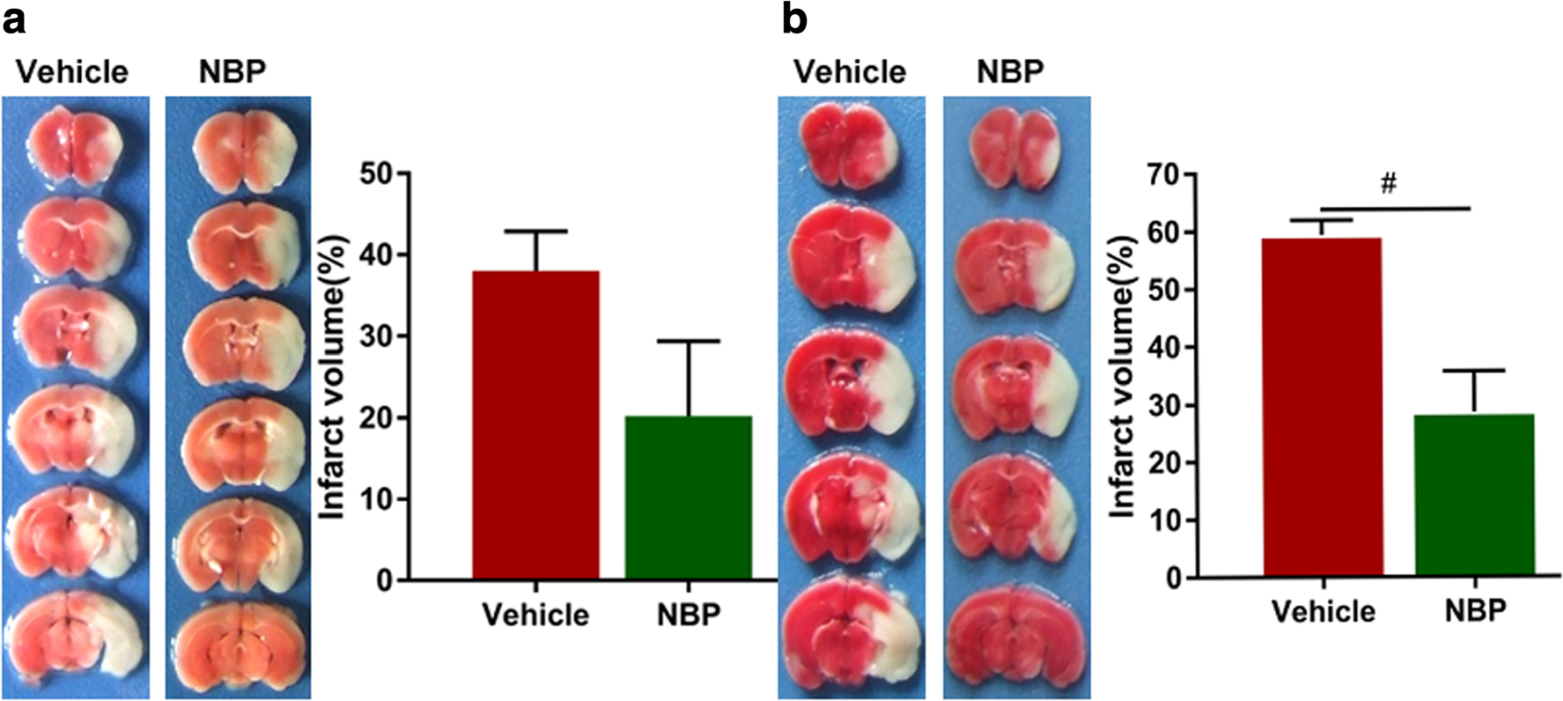
L-3-n butylphthalide (NBP) treatment significantly reduces infarct volume at 3 days after middle cerebral artery occlusion (MCAO). **a** Representative 2,3,5-triphenyltetrazolium chloride (TTC) staining at 1 day after MCAO. **b** Representative TTC staining at 3 days after MCAO. *N* = 5 animals per group. ^#^ *p* < 0.05 vs. vehicle-treated group

Unlike an earlier study, in which NBP was administrated intragastrically (Zhang et al. 2012), we administered NBP (14 mg/kg/day) via caudal vein immediately after reperfusion and then evaluated infarct volume at 1 and 3 days after MCAO. NBP treatment showed a tendency to decrease cerebral infarct volume at 1 day after MCAO reperfusion, albeit the difference was not significant (Fig. 1a, *p* = 0.11). However, a significant reduction in cerebral infarct volume was observed at 3 days after MCAO reperfusion (Fig. 1b, *p* < 0.05).

### NBP treatment significantly improves functional outcome in early phase after MCAO

**Fig. 2:**
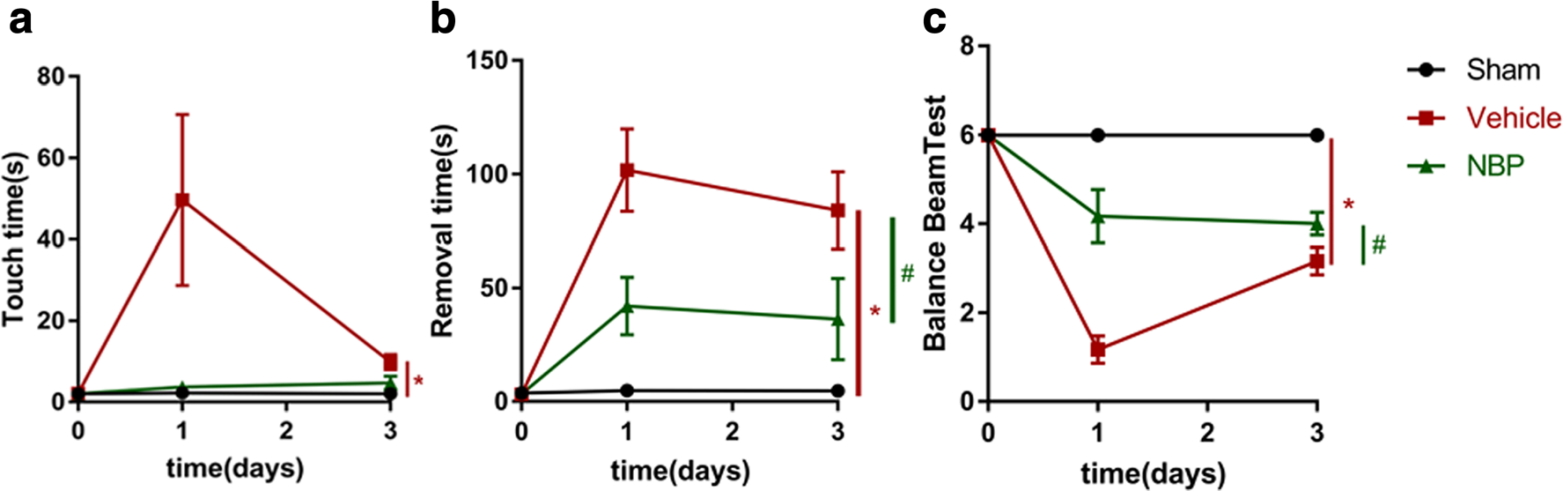
L-3-n butylphthalide (NBP) treatment significantly improves neurological function in early phase after middle cerebral artery occlusion (MCAO). Sensorimotor deficits were evaluated before MCAO, as well as 1 and 3 days after stroke using adhesive removal test and balance beam test. **a** Adhesive removal test: time to touch tape. **b** Adhesive removal test: time to remove tape. **c** Score of balance beam test. *N* = 6 animals per group. Data are presented as mean ± SEM. ^*^ *p* < 0.05 vs. sham group. ^#^ *p* < 0.05 vs. vehicle-treated group

Stroke results in sensory and motorial asymmetries, yet unlike previous research that used rats to study the effect of NBP on ischemia with Garcia test to assess behavioral abilities (Liu et al. 2007), we examined the effect of NBP on neurological function in mice before, as well as at 1 and 3 days after MCAO, using adhesive removal test and balance beam test. NBP treatment partially improved sensorimotor function after MCAO, as represented by a downward trend in time to touch the tape and a significant reduction in time to remove the tape in the adhesive removal test (Fig. 2a, b). Balance and motor coordination as evaluated by balance beam test also showed a significant improvement in NBP-treated group compared with vehicle-treated group (Fig. 2c, *p* < 0.05). These data suggest that NBP improves some aspects of neurological function in early phase following MCAO.

### Administration of NBP facilitates M2 polarization and suppresses M1 polarization of microglia in early phase after MCAO

**Fig. 3:**
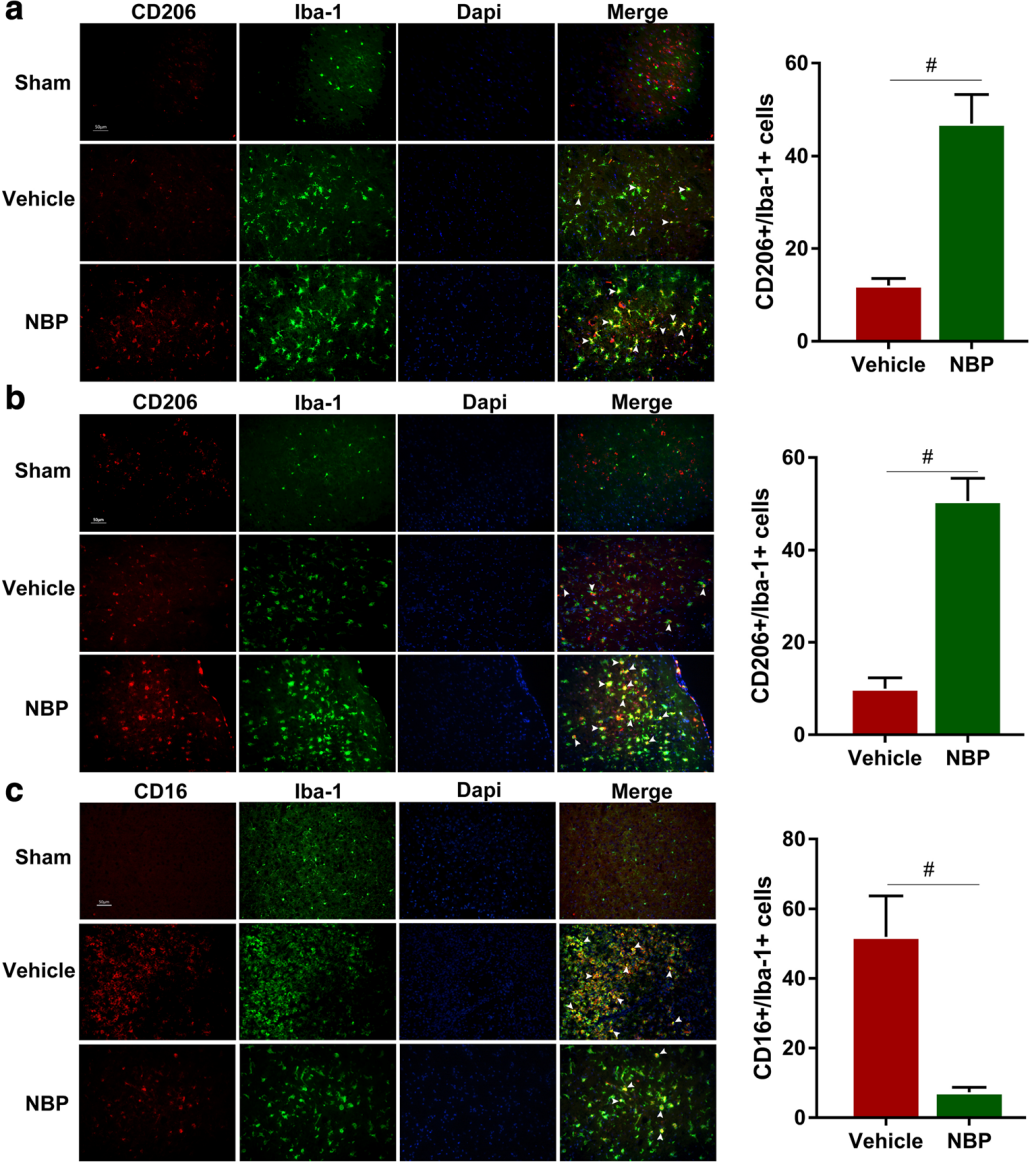
L-3-n butylphthalide (NBP) treatment significantly enhances M2 polarization and suppresses M1 polarization of microglia/macrophages in early phase after middle cerebral artery occlusion (MCAO). Representative double-immunofluorescence staining for CD206 (red) or CD16/32 (red) and Iba-1 (green) markers in cortex acquired from NBP or vehicle-treated mice at 1 or 3 days after MCAO, as well as sham-operated mice. **a** Double-immunofluorescence staining for CD206 (red) and Iba-1 (green) at 1 day after MCAO, white arrow represents CD206^+^/Iba-1^+^ double positive cells. **b** Double-immunofluorescence staining for CD206 (red) and Iba-1 (green) at 3 days after MCAO, white arrows represent CD206^+^/Iba-1^+^ double positive cell. **c** Double-immunofluorescence staining for CD16/32 (red) and Iba-1 (green) at 3 days after MCAO, white arrow represent CD16^+^/Iba-1^+^ double positive cell. *N* = 3 animals per group. Scale bar: 50 μm. Data are presented as mean ± SEM. ^#^ *p* < 0.05 vs. vehicle-treated group

Although both earlier studies and our own work here have shown that NBP is protective against ischemic stroke, its effect on microglial polarization remains unclear. The role of microglia/macrophages in the progression of cerebral ischemia has recently attracted increasing attention and microglia/macrophages are known to shift their phenotype from an early anti-inflammatory M2 phenotype to a later pro-inflammatory M1 phenotype after MCAO (Hu et al. 2012). Here, we explored whether administration of NBP influences microglial polarization by examining the expression of M1 markers (CD16/32) and M2 markers (CD206). Double immunofluorescence staining for CD16/32 or CD206 and Iba-1 (microglial marker) was performed at 1 and 3 days after MCAO. In sham-operated group, microglia showed non-activated with ramified morphology, while in vehicle-treated and NBP-treated groups microglia were obviously activated, featured by hypertrophic morphology with thickened processes. Moreover, the number of CD206^+^/Iba-1^+^ cells in NBP-treated group was significantly higher than that in vehicle-treated group at 1 day after MCAO (Fig. 3a, *p* < 0.05**)**. However, the number of CD16^+^/Iba-1^+^ cell remained almost unchanged (data not shown). Consistently, the number of CD206^+^/Iba-1^+^ cells in NBP-treated group remained higher than that in vehicle-treated group, while the number of CD16^+^/Iba-1^+^ cells showed a significant decrease at 3 days after MCAO (Fig. 3b, c, *p* < 0.05**)**. Taken together, our data suggest that NBP treatment facilitates M2 polarization and suppresses M1 polarization of microglia in early phase after MCAO, consistent with its neuroprotective function.

### NBP treatment protects against neuronal apoptosis at 7 days after MCAO

**Fig. 4:**
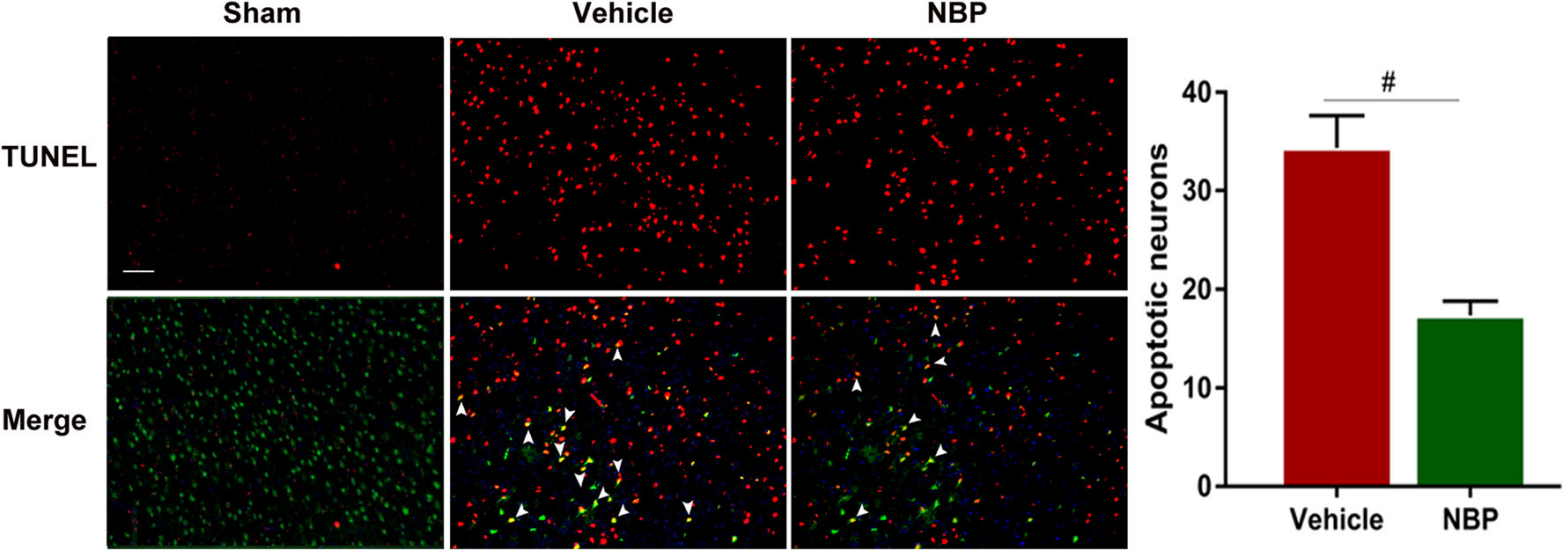
L-3-n butylphthalide (NBP) treatment protects against neuronal apoptosis at 7 days after MCAO. Representative TUNEL staining for neurons (green) and TUNEL (red) in cortex acquired from NBP or vehicle-treated mice at 7 days after MCAO, as well as sham-operated mice. Scale bar: 50 μm. White arrows represent apoptotic neurons. *N* = 3 animals per group. Data are presented as mean ± SEM. ^#^ *p* < 0.05 vs. vehicle-treated group

To further evaluate the protective effect of NBP, TUNEL staining was performed to detect the apoptotic neurons. There were few apoptotic neurons in sham-operated group, while in vehicle-treated group there were large quantities of apoptotic neurons, but this condition was largely reversed by NBP treatment (Fig. 4, *p* < 0.05). These results suggest that NBP treatment also protects against neuronal apoptosis in early phase after MCAO.

### Administration of NBP enhances M2 polarization and inhibits M1 polarization of microglia at 7 days after MCAO

**Fig. 5:**
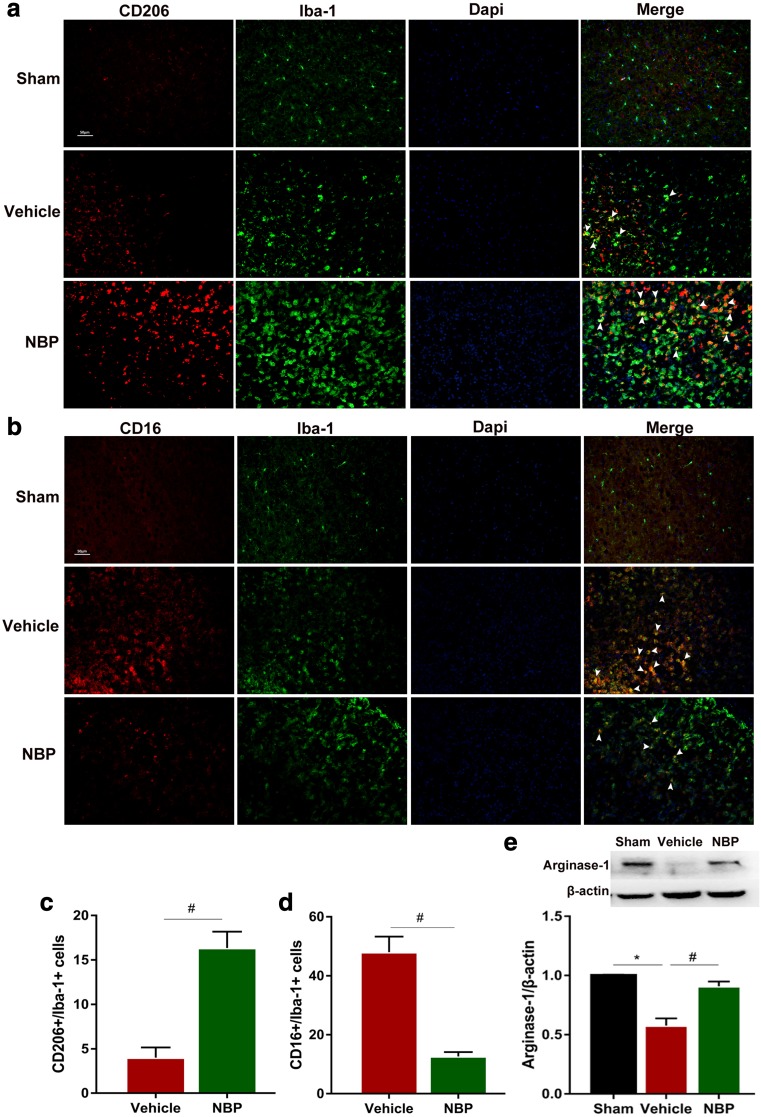
L-3-n butylphthalide (NBP) treatment significantly regulates microglia/macrophages towards M2 polarization at 7 days after MCAO. **a** Double-immunofluorescence staining for CD206 (red) and Iba-1 (green) at 7 days after middle cerebral artery occlusion (MCAO), white arrows represent CD206^+^/Iba-1^+^ double positive cells. **b** Double-immunofluorescence staining for CD16 (red) and Iba-1 (green) at 7 days after MCAO, white arrow represent CD16^+^/Iba-1^+^ double positive cells. Scale bar: 50 μm. **c** Quantitative analysis of CD206^+^/Iba-1^+^ cells in vehicle-treated group and NBP-treated group. **d** Quantitative analysis of CD16^+^/Iba-1^+^ cells in vehicle-treated group and NBP-treated group. **e** Arginase-1 protein expression measured by western blot analysis,and ration of arginase-1 in sham-operated group (black), vehicle-treated group (red) and NBP-treated group (green). *N* = 3 animals per group. Data are presented as mean ± SEM. ^*^ *p* < 0.05 vs. sham-operated group. ^#^ *p* < 0.05 vs. vehicle-treated group

A previous study suggests that M2 microglia/macrophages are detectable at 12 h, peaked at 1 to 3 days, and then declined several days after ischemic stroke, while M1 microglia/macrophages increased from day 3 to at least 14 days after stroke (Kanazawa et al. 2017). Thus, transforming microglia/macrophages from M1 state to M2 phenotype in the first 14 days after stroke seems to be beneficial. To further evaluate the effect of NBP on microglia/macrophages polarization, we performed immunofluorescence staining and western blot to determine whether NBP influenced microglia polarization at 7 days after MCAO. Immunofluorescence staining showed that NBP administration substantially increased the number of CD206^+^/Iba-1^+^ cells compared with vehicle-treated mice, while the number of CD16^+^/Iba-1^+^ cells showed a constant decrease at 7 days after MCAO (Fig. 5a-d, *p* < 0.05**)**. Similarly, western blot showed that expression of M2 marker, arg-1 was markedly increased in NBP-treated group compared with vehicle-treated group (Fig. 5e, *p* < 0.05). Collectively, these findings demonstrate that NBP treatment enhances M2 polarization and inhibits M1 polarization of microglia/macrophages in early phase of ischemic stroke, last at least 7 days.

## Discussion

Microglia/macrophages are highly plastic cells that respond sensitively to ischemic stroke and transit rapidly between different phenotypes (Kanazawa et al. 2017). Many recent studies have demonstrated that promoting microglia/macrophages polarization from M1 to M2 state enhances recovery after stroke. In the present study, our findings show that NBP significantly reduces infarct volume and attenuates neurological impairment. We also find there is a significant M1-to-M2 transformation after NBP treatment. Thus, we conclude that the neuroprotective effect of NBP is likely mediated by changes in microglia/macrophages polarization. To our knowledge, this is the first study to demonstrate that NBP may exert its neuroprotective effect by regulating microglia/macrophages polarization.

Neuroinflammation is a crucial mechanism in the pathogenesis of ischemic stroke, and brain-intrinsic microglia and recruited macrophages are the first immune cells respond to ischemic insult (Chamorro et al. 2016). When ischemic stroke occurs, microglia/macrophages shift their phenotype from the downregulated status to the activated phenotype, featured by hypertrophic morphology, with extensively branched and retracted processes (Qin et al. 2017). Activated microglia/macrophages are roughly divided into two types by recent researches. “Classically activated” M1 microglia/macrophages, which have reduced phagocytic capacity and increased secretion of destructive pro-inflammatory mediators, are thought to be harmful; and “alternatively activated” M2 microglia/macrophages, which show enhanced phagocytic activity and release a plethora of neurotrophic/protective factors, are believed to be a healthier phenotype (Xia et al. 2015). While broad suppression or depletion of microglia/macrophages may exacerbate brain injury (Perego et al. 2016; Wang et al. 2013a), an increasing number of studies suggest that a preferred therapeutic option is to promote a M1 to M2 switch or to balance these two phenotypes rather than bring about blanket suppression of microglia/macrophages.

NBP is a natural product originally extracted from celery seeds, and has remarkable neuroprotective effects against cerebral ischemia (Cui et al. 2013; Hu et al. 2014; Zhao et al. 2014), as well as other neurological diseases including vascular dementia, diffuse brain injury, amyotrophic lateral sclerosis, Alzheimer’s disease and autoimmune encephalomyelitis (Feng et al. 2012; Huai et al. 2013; Wang et al. 2018a; Zhang et al. 2016b; Zhao et al. 2013). Although an increasing number of studies suggest that NBP may protect against ischemic stroke through several signaling pathways (Wen et al. 2016; Xu et al. 2017; Yan et al. 2017), the effect of NBP on microglia polarization has not been elucidated. Consistent with previous reports, we first showed that treatment with NBP not only reduced cerebral infarct volume, but also boosted neurological performance in early stage after ischemic stroke. We then explored the effect of NBP on polarization of microglia/macrophages and found that treatment with NBP markedly promoted microglia/macrophages polarization towards M2 phenotype at 1 and 3 days after MCAO, as evidenced by a decrease of CD16^+^/Iba-1^+^ cells and an increase of CD206^+^/Iba-1^+^ cell. Consistent with this, previous studies have suggested that modulating polarization of microglia/macrophages led to a neuroprotective effect in ischemic stroke models (He et al. 2017; Li et al. 2016; Wang et al. 2018b). Taking into account our own results and those of previous studies, we conclude that NBP has a protective effect in ischemic stroke, likely by skewing microglia/macrophages polarization from an M1 phenotype to an M2 phenotype.

Since NBP has a neuroprotective effect in ischemic stroke and switching microglia/macrophages from an M1 to an M2 phenotype seems to be beneficial for the recovery of ischemic stroke, we also investigated whether NBP promotes neuronal survival and influences microglia/macrophage polarization at 7 days after MCAO. Using TUNEL staining, we found that NBP treatment significantly reduced neuronal apoptosis compared with vehicle-treated mice. An earlier study showed that neuronal dysfunction and death may cause release of pro-inflammatory mediators by microglia (Loane and Byrnes 2010). Our results further suggest that NBP may exert neuroprotective effects by reducing the release of pro-inflammatory factors from M1 microglia/macrophages. Immunofluorescence staining showed that NBP reduced the number of M1 microglia/macrophages and upregulated M2 microglia/macrophages. Based on these findings, we believe that NBP may protect against cerebral ischemia by switching M1 microglia/macrophages to an M2 phenotype and inhibiting neurons death.

Although the underlying mechanism about how NBP enhances microglia/macrophages polarization towards M2 phenotype is not yet clear, several signaling pathways involved in microglia polarization maybe activated by NBP. For example, it has been suggested that NBP activates Akt kinase pathway (Qi et al. 2017; Xiang et al. 2014), which is considered important in polarizing microglia toward M2 phenotype (Wang et al. 2015). NBP has also been shown to attenuate inflammatory responses in cultured astrocytes through nuclear factor-κB (NF-κB) pathway (Wang et al. 2013a), and NF-κB is suggested to be involved in suppressing the activation of M1 microglial (Park et al. 2012). In addition, NBP could protect against ischemia-induced brain damage by inhibiting the c-Jun N-terminal kinase (JNK) signaling pathway (Wen et al. 2016), and coincidently, JNK phosphorylation may participate in the modulation of M1-to-M2 polarization (Xiang et al. 2018). Further studies are needed to determine the precise mechanisms by which NBP protects against ischemic stroke and influences microglia/macrophages polarization.

## Conclusion

In conclusion, in the present study we demonstrate that NBP reduces cerebral infarct volume and attenuates neurological impairment in early phase after MCAO, likely by switching microglia/macrophages from the detrimental M1 phenotype to the protective M2 phenotype. Accordingly, we believe that NBP may provide a promising treatment for patients with acute ischemic stroke, and that the mechanisms underlying the switch in microglia/macrophages polarization are worthy of further evaluation.
